# Myocardial infarction with nonobstructive coronary arteries due to acute coronary vasospasm induced by toripalimab: a case report and review of literature

**DOI:** 10.3389/fcvm.2025.1646968

**Published:** 2025-09-01

**Authors:** Bihan Huang, Shaoyuan Chen, Haigang Zhang, Xueying Han

**Affiliations:** ^1^ Department of Cardiology, Shenzhen Nanshan People’s Hospital, Shenzhen, China; ^2^ Department of Intensive Care, Shenzhen Nanshan People’s Hospital, Shenzhen, China

**Keywords:** MINOCA, coronary artery spasm, acute myocardial infarction, PD-1, toripalimab

## Abstract

**Background:**

Coronary artery spasm (CAS), one of the etiologies for MINOCA, is an uncommon cause of acute chest pain. Toripalimab, a recombinant monoclonal antibody targeting programmed death receptor 1 (PD-1), exhibits a wide range of anti-tumor activities. Nevertheless, instances of toripalimab-induced cardiotoxicity have been seldom reported.

**Methods:**

We present the case of a 60-year-old male patient diagnosed with hepatocellular carcinoma who experienced MINOCA subsequent to the administration of toripalimab. Based on the patient's symptoms, electrocardiogram (ECG) findings, and coronary angiography, transient occurrence CAS was established. The patient was prescribed diltiazem sustained-release capsules. During his follow-up on an outpatient basis, he did not experience a recurrence of the previously reported chest discomfort or any other symptoms. We used the CARE checklist when writing our report.

**Conclusion:**

This is the first case report of MINOCA induced by toripalimab, mediated through coronary artery spasm. This case report emphasizes the awareness regarding the potential for severe cardiovascular complications associated with the administration of toripalimab.

## Introduction

Myocardial Infarction with No Obstructive Coronary Artery disease (MINOCA) refers to patients who meet the criteria for a fourth myocardial infarction but do not have any obstructive coronary artery disease (≥50% stenosis) on coronary angiography (1–3). MINOCA can be caused by various factors, including coronary artery spasm (CAS) syndrome, coronary dissection, *in situ* thrombosis, tachycardia, and coronary embolism (4). Toripalimab, a humanized IgG4K monoclonal antibody specifically targeting human PD-1, is the first domestically marketed anti-tumor PD-1 antibody in China (5). It has demonstrated a wide spectrum of anti-tumor activity, including melanoma, lung cancer, and gastrointestinal tumors (6–8). However, it is uncommon for toripalimab to cause acute coronary vasoconstriction. This case report documents an instance of acute ST-segment elevation myocardial infarction caused by severe coronary vasospasm following the administration of toripalimab in a 60-year-old man.

## Case presentation

**Figure 1:**
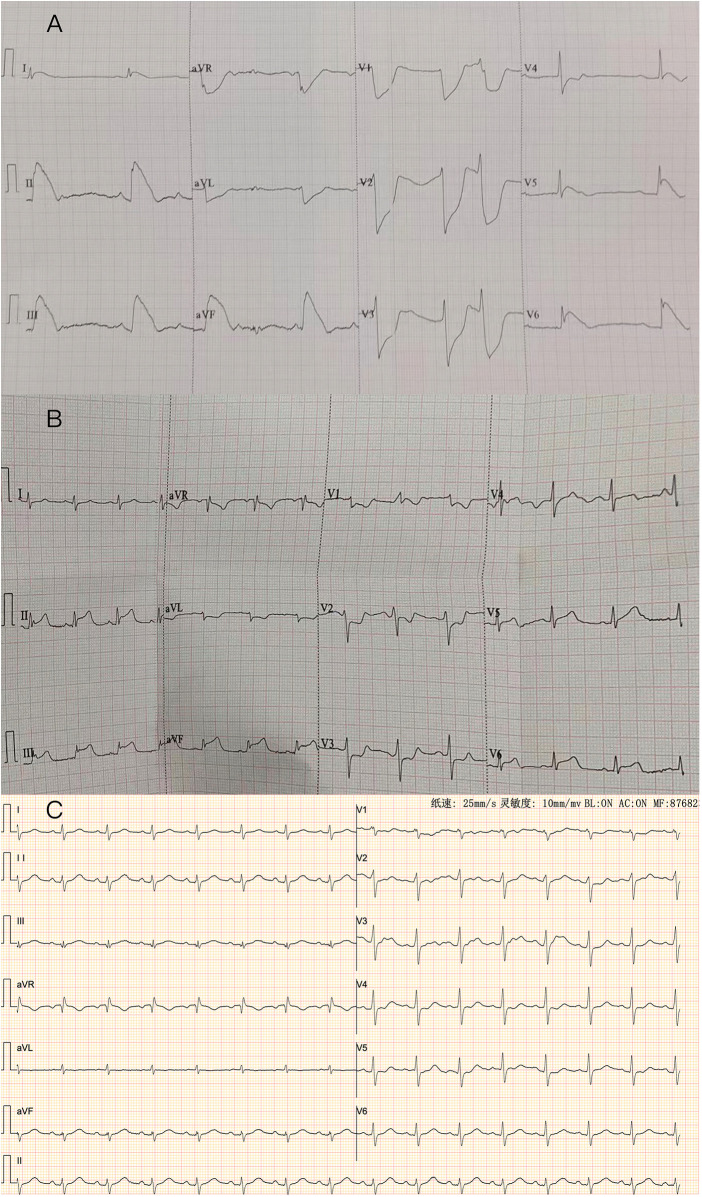
**(A)** ECG: the ECG shows a 0.1–1.0 mv ST elevation at leads DI, II, III, aVF, V5, V6, with reciprocal depression in DaVL, V1, V2, V3 and V4. **(B)** ECG: The ECG shows a 0.1–0.2 mv ST elevation at leads DII, III, aVF, V5, V6, with reciprocal depression in DI, aVL, V1, V2, and V3. **(C)**: Repeated ECG revealed normal sinus rhythm with no significant ST-T changes in all leads.

**Figure 2:**
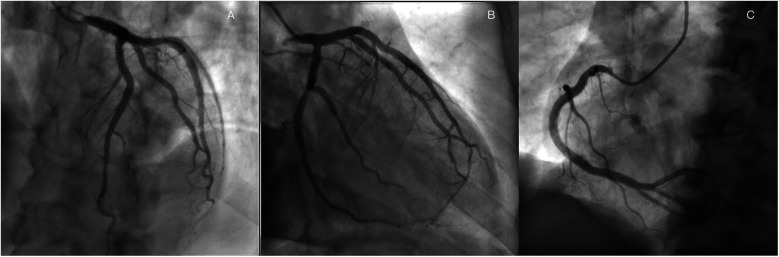
Coronary angiography revealed nonobstructive coronary arteries in the LAD, **(A)**, LCX, **(B)** RCA **(C).**

A 60-year-old man was found to have a liver-occupying lesion during a medical checkup. He underwent laparoscopic liver resection of segment 7 and was diagnosed with hepatocellular carcinoma (HCC) via liver biopsy in May 2022. Subsequently, he was initiated on a combination therapy involving tislelizumab (200 mg q3w) and lenvatinib (8 mg qd) from June 2022. Throughout the tislelizumab and lenvatinib combination therapy, no significant complications were observed. An MR scan of the upper abdomen conducted three months earlier showed a rounded signal in the S6 segment of the liver, along with adjacent multiple abnormal signal nodules. On May 4, 2023, the patient was admitted to the hepatobiliary surgery department. No chest discomfort was reported, and his troponin level was negative. His temperature was 36.4°C, pulse rate 81 bpm, respiratory rate 20 breaths per min, and blood pressure 124/79 mmHg. Cardiac and pulmonary examinations revealed normal heart sounds without murmurs, as well as clear lung sounds. Abdominal examination indicated a soft and nontender abdomen without rebound tenderness. A treatment plan was established for anti-tumor therapy, involving toripalimab and lenvatinib/oxaliplatin/gemcitabine. However, shortly after the infusion of toripalimab, the patient experienced sudden chest pain, diaphoresis, and then syncope. Upon physical examination, his heart rate dropped to 50 beats per min, respiratory rate remained at 20 breaths per minute, and blood pressure decreased to 85/60 mmHg. Auscultation indicated normal heart sounds and no murmurs. The patient's face appeared pale, with no rash. The electrocardiogram (ECG) displayed ST-segment elevation of 0.1–1.0 mV in dynamic leads I, II, III, aVF, and V5–V6 (Figure 1A). Toripalimab was promptly discontinued, leading to the spontaneous resolution of symptoms after 10 min and a return of the ECG to baseline. Cardiac troponin I enzyme levels peaked at 0.080 ng/ml (0–0.034) around 18 h after symptom onset. The patient did not agree to undergo coronary angiography (CAG) due to the symptom relief. Coronary computed tomographic angiography revealed nonobstructive coronary arteries with mild stenosis, 11% stenosis in the left main artery (LM), 11% stenosis in the proximal-left anterior descending artery (pLAD), myocardial bridge-mural coronary artery in the proximal-left anterior descending artery and the distal-left anterior descending artery (dLAD), without stenosis in the left circumflex artery (LCX), 10% stenosis in the middle right coronary artery (mRCA), and 10% stenosis in the distal right coronary artery (dRCA). A transthoracic echocardiogram confirmed normal left ventricular size and function. Lipid-regulating drugs atorvastatin (20 mg/day) was prescribed, and the patient remained symptom-free in the subsequent days. Thus, he was planned to continue to implement a anti-tumor therapy course. However, on May 25, 2023, during toripalimab infusion, the patient experienced a recurrence of chest pain, diaphoresis, and dizziness. Physical examination indicated a heart rate of 68 bpm, respiratory rate of 26 breaths per min, and blood pressure of 63/40 mmHg. Auscultation indicated normal heart sounds and no murmurs. The ECG revealed dynamic ST-segment elevation of 0.1–0.2 mV in leads II, III, aVF, and V5–V6 (Figure 1B). Toripalimab was immediately discontinued, leading to symptom resolution within 30 min, and the ECG returned to baseline (Figure 1C). Cardiac troponin I enzyme levels were measured at 0.138 ng/ml. A transthoracic echocardiogram revealed an ejection fraction of 60%, with normal left ventricle's size and no significant regional wall motion abnormalities. Following the doctor's advice, he had CAG examination. Subsequent CAG confirmed patent coronaries, without any substantial flow-limiting lesions (Figure 2). The patient did not agree to undergo coronary vasospasm provocation testing due to possible serious adverse event risk, and refused further examination to further confirm the cause. However, based on the patient's symptoms, ECG findings, and coronary angiography, a diagnosis of CAS was established. The patient was prescribed diltiazem sustained-release capsules (30 mg, four times daily) and atorvastatin (20 mg/day).

He was transferred back to the former department to complete the anti-tumor therapy after being stable. Throughout the subsequent anti-tumor treatment, the patient did not experience any chest pain or discomfort. Moreover, during his follow-up on an outpatient basis, he did not experience a recurrence of the previously reported chest discomfort or any other symptoms. Patient's timeline is shown in Table 1.

**Table 1:** Timeline.

Background	Hepatocellular carcinoma
Day zero (admission)	Infusion of toripalimab. Chest pain, diaphoresis, and then syncope. ST-segment elevation of 0.1–1.0 mV in leads I, II, III, aVF, and V5–V6. Elevation of troponin I. Coronary computed tomographic angiography revealed nonobstructive coronary arteries.
Day 3 weeks	Toripalimab infusion. Recurrence of chest pain, diaphoresis, and dizziness. ST-segment elevation of 0.1–0.2 mV in leads II, III, aVF, and V5–V6. Elevation of troponin I. Coronary angiography with absence of obstructive coronary arteries disease. Discharge after the patient was prescribed diltiazem sustained-release capsules (3 mg, four times daily) and atorvastatin.

## Discussion

Roughly 6% of patients presenting with acute myocardial infarction are identified as having non-obstructive coronary arteries through coronary angiography. The initial diagnosis of MINOCA requires the presence of ischemic signs/symptoms, an elevation and subsequent decline in cardiac troponin levels, with at least one value exceeding or equaling the 99th percentile upper limit of normal, and the identification of non-obstructive coronary artery disease through imaging techniques (9). Coronary vasospasm is characterized by a significant constriction of the coronary arteries, leading to a notable imbalance between oxygen demand and supply. This phenomenon can potentially induce substantial myocardial ischemia, acute myocardial infarction, or even sudden cardiac death (10). The primary treatment approach, calcium channel blockade, aims to diminish intracellular calcium levels in order to restrict contraction and spasm of coronary smooth muscle. Several pathological mechanisms have been proposed, including the direct impact of catecholamines, inflammation, endothelial cell dysfunction, hypercontractility of smooth muscle cells, or heightened oxidative stress (10). In contrast to typical angina arising from atherosclerotic artery disease, vasospastic angina resulting from coronary vasospasm frequently occurs in patients without notable cardiovascular risk factors (11). Furthermore, common triggers for this condition lncluede cold exposure, mental stress, stimulants, and medications (10, 11).

The advent of immune checkpoint inhibitors (ICIs) has brought about a transformative shift in cancer therapy. Among these ICIs, PD-1 plays a critical role in facilitating tumor immune evasion and modulating the immune response to identify and combat cancer cells (12). Immune-related adverse events (irAEs) are a result of the immune system being abnormally stimulated in normal tissues. Compared to irAEs in other organs, cardiac irAEs are relatively uncommon, but they have a significant mortality rate. Cardiac irAEs include myocarditis, pericarditis, heart failure, and myocardial infarction (13–16). Toripalimab, a relatively new ICIs developed in China, was usually well tolerated in clinical studies in Chinese patients with advanced malignancies.

Rare instances of toripalimab-related fatal cardiac irAEs have been reported. In this context, we present a case of a patient with HCC who experienced fatal cardiac irAEs subsequent to toripalimab therapy. To date, there have been only three case reports describing coronary spasm after administration of PD-L1 inhibitor (17–19). To the best of our knowledge, this is the first report to describe a patient who developed MINOCA due to coronary artery spasm induced by toripalimab.

A case of coronary artery spasm was caused by nivolumab (17). In a separate case, a patient undergoing pembrolizumab treatment, a PD-L1 inhibitor, experienced coronary artery spasm (18). It was attributed to a systemic inflammatory reaction, and the patient's condition rapidly improved following anti-inflammatory and immunomodulatory treatments. In the third case, thyrotoxicosis-associated coronary artery spasm and ventricular tachycardia were prompted by camrelizumab, a PD-1 inhibitor (19). Mechanisms behind thyroid hormone-induced coronary spasm encompass an imbalance between blood supply and oxygen demand during thyrotoxic states, coupled with augmented vascular reactivity (20). In contrast, our patient's coronary artery spasm was neither a consequence of ICIs-associated vasculitis nor thyrotoxicosis, given the absence of hyperthyroidism, the lack of administration of immunosuppressive or anti-inflammatory medications and calcium channel blockers were efficacive.

Kounis syndrome, also known as hypersensitivity acute coronary syndrome, is a rare disorder in which an allergic or hypersensitivity reaction triggers an acute coronary syndrome. Type I Kounis syndrome is usually associated with MINOCA, in which the coronary arteries are normal or nearly normal (21).

The exact cause of coronary artery spasm induced by ICIs remains not fully elucidated. One mechanisms suggests that immunotherapy could directly exert toxicity on cardiac cells through PD-1 inhibition. Both cardiomyocytes and vascular endothelial cells might express programmed death-ligand 1 (PD-L1). When ICIs prevent the interaction between PD-1 and PD-L1, endothelial cells become susceptible to attack by CD8+ T lymphocytes, which execute endothelial cell destruction through perforin-mediated cytolysis. This intensified cytolytic activity of CD8+ T lymphocytes further compromises vascular integrity, leading to vascular injury (22). Additionally, a number of studies employing human and animal models have demonstrated that a deficiency in PD-1/PD-L1 exacerbates the progression of atherosclerotic plaques and raises levels of CD4+ and CD8+ *T* cells, and anti-PD-1 antibodies thave a propensity to heighten inflammation (23–25). These mechanisms can contribute to vascular spasms (10).

In conclusion, this case analysis suggests that ICIS use is associated with MINOCA, although we do not yet have conclusive evidence to elucidate the mechanism. Future studies should investigate the mechanisms by which ICIs lead to myocardial infarction. This case report emphasizes the awareness regarding the potential for severe cardiovascular complications associated with the administration of toripalimab.

## Conclusion

To our understanding, this is the first case report of a toripalimab-induced MINOCA due to acute coronary vasospasm. Monitoring is required during therapy to guarantee patient safety. Given the common exclusion of cancer patients from randomized controlled trials, the management experience for this population was also limited to observational research. Therefore, we urgently require additional clinical experience and research data to aid in the development of treatment strategies.

## Data Availability

The original contributions presented in the study are included in the article/Supplementary Material, further inquiries can be directed to the corresponding author.
